# Disturbing-Free Determination of Yeast Concentration in DI Water and in Glucose Using Impedance Biochips

**DOI:** 10.3390/bios10010007

**Published:** 2020-01-19

**Authors:** Mahdi Kiani, Nan Du, Manja Vogel, Johannes Raff, Uwe Hübner, Ilona Skorupa, Danilo Bürger, Stefan E. Schulz, Oliver G. Schmidt, Daniel Blaschke, Heidemarie Schmidt

**Affiliations:** 1 Department Back-End of Line, Fraunhofer Institute for Electronic Nano Systems, Technologie-Campus 3, 09126 Chemnitz, Germany; danilo.buerger@enas.fraunhofer.de (D.B.); stefan.schulz@enas.fraunhofer.de (S.E.S.); 2 Institute for Solid State Physics, University of Jena, 07743 Jena, Germany; 3 Leibniz Institute of Photonic Technology, Albert-Einstein-Str. 9, 07745 Jena, Germany; uwe.huebner@leibniz-ipht.de (U.H.); daniel.blaschke@leibniz-ipht.de (D.B.); 4 Helmholtz-Zentrum Dresden-Rossendorf, Bautzner Landstraße 400, 01328 Dresden, Germany; m.vogel@hzdr.de (M.V.); j.raff@hzdr.de (J.R.); i.skorupa@hzdr.de (I.S.); 5 Center for Microtechnologies, Chemnitz University of Technology, Reichenhainer Str. 70, 09126 Chemnitz, Germany; 6 Institute for Integrative Nanosciences IFW Dresden, Helmholtzstr. 20, 01069 Dresden, Germany; o.schmidt@ifw-dresden.de

**Keywords:** biochips, impedance spectroscopy, yeast *Saccharomyces cerevisiae*, electrical equivalent circuit, biomaterial, biosensing

## Abstract

Deionized water and glucose without yeast and with yeast (*Saccharomyces cerevisiae*) of optical density OD_600_ that ranges from 4 to 16 has been put in the ring electrode region of six different types of impedance biochips and impedance has been measured in dependence on the added volume (20, 21, 22, 23, 24, 25 µL). The measured impedance of two out of the six types of biochips is strongly sensitive to the addition of both liquid without yeast and liquid with yeast and modelled impedance reveals a linear relationship between the impedance model parameters and yeast concentration. The presented biochips allow for continuous impedance measurements without interrupting the cultivation of the yeast. A multiparameter fit of the impedance model parameters allows for determining the concentration of yeast (c_y_) in the range from c_y_ = 3.3 × 10^7^ to c_y_ = 17 × 10^7^ cells/mL. This work shows that independent on the liquid, i.e., DI water or glucose, the impedance model parameters of the two most sensitive types of biochips with liquid without yeast and with liquid with yeast are clearly distinguishable for the two most sensitive types of biochips.

## 1. Introduction

There is an ongoing search towards disturbing-free determination of biomaterial concentration, e.g., to gain better control over the growth of cell cultures. Standard optical microscopy investigations disturb cell growth. Therefore, this method is less convenient and time consuming (not suitable for long term measurements and number determination).

Nowadays, optical density (OD) is measured to estimate the growth of cell cultures. The challenge is to maximize “signal to noise” ratio and find a large range of measurement data with a linear relationship between measurement data and biomaterial concentration. OD data reveal a linear dependence on the concentration of biomaterial only in a limited range. The presented disturbing–free determination of the cell density that is based on the impedance spectroscopy promises numbers of advantages when compared to the other common techniques of cell density/number detection. The plate count agar (PCA) technique and counting under microscope would be highly time consuming and it always involves human errors. Such disadvantages call for more adequate electrical analysis methods, e.g., microfluidic impedance cytometer [1,2,3,4] and impedance biochips, which have been used to count and discriminate yeast cells on the basis of their dielectric and electrical properties. The determination of the cell density with impedance biochips, on the contrary, is extremely fast and it prevents operator malfunctions. Impedance chips that are made of highly doped silicon have been prepared by depositing lipid membranes on highly doped silicon as top electrodes. The contact resistance of those lipid membranes in dependence on the formation of transmembrane pores has been detected from impedance measurements in the 10 mHz to 20 kHz range [5]. Additionally, lipid terminated polyelectrolytes have been deposited on n-type Si with a native SiO_2_ and impedance has been measured from 100 mHz to 100 kHz [6] to analyze the lipid polyelectrolyte interaction.

In perspective applications, the impedance biochips will be developed for the determination of cell concentration in a large range of concentration, where sensitivity of other methods, e.g., of OD measurements is typically limited (OD < 0.1 and OD > 1.5). In this work, we performed detailed investigation in the large cell concentration range from c_y_ = 3.3 × 10^7^ to c_y_ = 17 × 10^7^ cells/mL, which corresponds to a linearly extrapolated OD that ranges from 4 to 16. 

In a previous work, two types of novel P-N junction-based Si biochips have been presented and a bacteria suspension, i.e., *Lysinibacillus* sphaericus JG-A12, in Deionized (DI) water, with an extrapolated OD_600_ that ranges from 4 to 16 has been filled in the ring electrode area of the impedance biochips. It has been shown that the measured impedance is significantly different and systematically changing if 1,2,3,4, and 5 µL pure DI water is added to 20 μL DI water or if 1, 2, 3, 4, and 5 µL of the bacteria suspension is added to the 20 μL DI water [7]. In the present work, yeast *Saccharomyces cerevisiae* (*S. cerevisiae*), suspended in two different liquids, namely DI water and glucose, and with an extrapolated OD_600_ ranging from 4 to 16 has been filled in the ring electrode area of six different types of the novel P-N junction-based Si biochips. Yeast *S. cerevisiae* is a model organism in molecular and cell biology. It is a facultative anaerobic microorganism that has been used by mankind for several thousand years because of its high fermentative capacity to convert sugar into ethanol and CO_2_.

This leads to the production of alcoholic beverages and dough leavening for baking bread [8]. In biotechnology, *S. cerevisiae* was the first eukaryote to be used for recombinant protein production, e.g., human interferon [9] or insulin [10]. Additionally, *S. cerevisiae* was the first eukaryote to have its genome fully sequenced. This placed *S. cerevisiae*, or a long time, at the leading edge of genomic-scale research, including microarrays, systematic gene deletion, and, more recently, the construction of a fully synthetic eukaryotic genome. A wide range of commercially available collections and a dedicated database make it easy for new researchers to use this system to answer fundamentally important questions in eukaryotic cell biology [11]. We use six types of impedance biochips and investigate the impedance change of the biochips if 1,2,3,4, and 5 μL DI water and glucose is added to 20 μL DI water and glucose, respectively, and if 1,2,3,4, and 5 μL suspension of yeast (*Saccharomyces cerevisiae*) with optical density OD_600_ that ranges from 4 to 16 in DI water or glucose is added to 20 μL DI water or glucose, respectively. The manuscript is structured, as follows: in Section 2, we present the six types of impedance biochips, in Section 3, measured and modeled impedance data are presented. The results are discussed in Section 4 and the paper is closed by the conclusion in Section 5.

## 2. Materials and Methods

*S. cerevisiae* cells used in this research were cultivated overnight in 50 mL Erlenmeyer flasks in sterile medium (yeast extract 3 g/L, malt extract 3 g/L, peptone 5 g/L, glucose 10 g/L) under shaking (100 rpm) at 30 °C. The cell density was determined by measuring the optical density at 600 nm (OD_600_) while using a UV-Vis spectrometer specord 50 (HZDR, Dresden, Germany). Prior to use, the yeast cells were harvested by centrifugation, washed two times with DI water, and re-suspended in DI water giving an OD_600_ of 80. Correlation between OD_600_ and cell number was achieved by cell counting under a microscope while using a Neubauer counting chamber.

### 2.1. Structural Description of the Biochips

**Figure 1:**
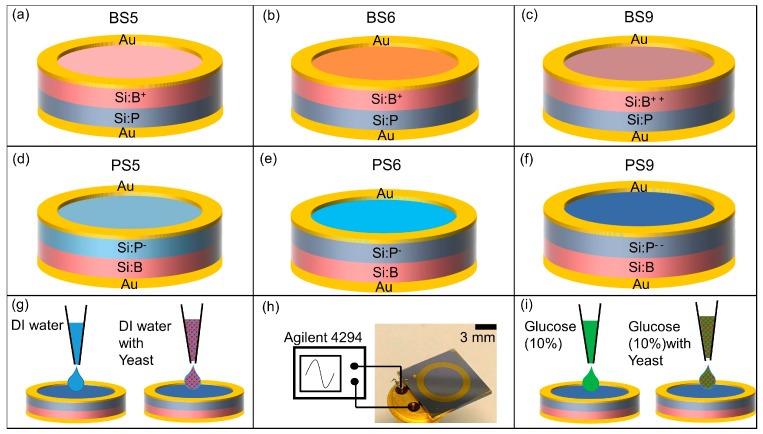
Schematic sketch of the p-n junction-based Si biochip with a ring top electrode with (**a**–**c**) boron ions (B+) implanted into Si:P or with (**d**–**f**) phosphorous (P-) ions implanted into Si:B. BS6 (**b**) and PS6 (**e**) are annealed in nitrogen for 2 h. BS9 (**c**) and PS9 (**f**) are highly doped biochips. Top and bottom electrodes have been wire bonded to the pins of a TO-5 package (**h**) and connected to an Agilent 4294 A impedance analyzer. DI water (**g**) and *S. cerevisiae* suspension or 10% glucose as medium with *S. cerevisiae* (**i**) are added in to the top ring electrode for impedance spectroscopy.

Six different types of biochips have been prepared for investigating the impedance variation of the biochip in dependence on the cell concentration (Table 1). The dopant types and annealing condition differ in these biochips. Biochips BS6 and PS6 are annealed for 2 h in nitrogen at 900 °C. Table 1 lists the overview of the implantation parameters for the manufacturing of the biochips with ring top electrodes. As depicted in Figure 1, for fabricating the BS biochips, Boron ions (B+) implanted into Si:P and for creating the PS biochips, phosphorous (P-) ions implanted into Si:B. The 150 nm thick gold (Au) ring top electrodes have been deposited by dc-magnetron sputtering with inner and outer diameters of 5.7 mm and 7.8 mm on silicon wafers of thickness 525 µm and unstructured gold (Au) creates the bottom contact. The sensitivity of the six types of biochips has been studied as a function of the ion fluence. Two different groups of biochips are fabricated, one with standard doping density and other group with high doping density. In phosphor standard-doped biochips (PS5, PS6,BS5, BS6), phosphorous ions with ion fluence of 3 × 10^13^ cm^−2^ have been implanted to the P-type Si wafers, whereas phosphorous ions with ion fluence of 3 × 10^15^ cm^−2^ have been implanted into the P-type Si wafers for the high doped biochips (PS9). In the standard boron-biochip (BS5, BS6) boron ions have been implanted into a N-type Si wafer with ion fluence of 3 × 10^13^ cm^−2^ and for the high doped biochips (BS9) boron ions with ion fluence of 8 × 10^15^ cm^−2^ have been implanted in N-type Si wafers. 

A ring electrode has been chosen because of the homogenous field distribution between the top and bottom electrodes. The limit of detection of the Si biochip strongly depends on the volume of liquid for which the top electrode structure is optimized. The presented ring electrode structure is optimized with respect to ring inner and outer diameter for sensing volumes between 10 and 30 μL. The inner and outer diameter of the ring electrode should be reduced if a smaller number of biomaterial should be detected.

The top and bottom contacts of the biochips are bonded to the standard TO-5 package (Figure 1i). The impedance characteristics of biochips, then, have been recorded within the frequency range from 40 Hz to 1 MHz under normal daylight at room temperature by using the Agilent 4294A precision impedance analyzer.

**Figure 2:**
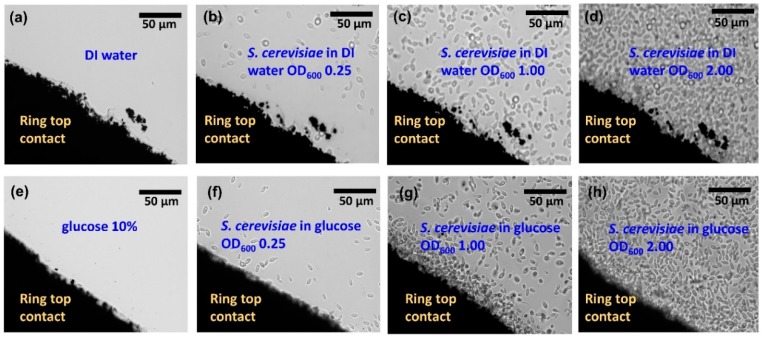
Top view optical microscopic images of a section of ring electrode (dark)on glass (bright) with (**a**) DI water, (**e**) glucose, *S. cerevisiae* in (**b**) DI water, (**f**) glucose at OD_600_ 0.25, *S. cerevisiae* in (**c**) DI water (**g**) in glucose at OD_600_ 1.00, *S. cerevisiae* in (**d**) DI water and (**h**) in glucose at OD_600_ 2.00 in the ring top electrode region. Here a transparent glass substrate has been used to illuminate the sample with light from the backside. The thickness of the ring top electrodes is 150 nm and is large enough to keep inserted liquid in the ring top electrode.

This impedance analyzer works in the range from 10^−3^ to 10^8^ Ohm and it is suitable for impedance spectroscopy on the investigated Si biochips with the impedance change in the range up to 10^5^ Ohm. In the impedance experiments, either solvent DI water with the *S. cerevisiae* (Figure 1g) or solvent glucose (10%) with the *S. cerevisiae* (Figure 1i) have been added into the Au ring top electrode region. The optical microscopy images with two different solvents i.e., DI water (Figure 2a–d) and glucose (Figure 2e–h) have been taken before adding (Figure 2a) and after adding *S. cerevisiae* with corresponding optical density at 600 nm (OD_600_) in order to visualize the different concentrations of *S. cerevisiae*.

For taking these images, the Au ring top electrodes have been deposited on a glass slide for utilizing the phase contrast mode of the microscope. For microbial cell density, the Optical OD_600_ is a common measure, which can be correlated to the cell number per volume, depending on the chosen biomaterial. In this work, the OD_600_ of four up to 16 are applied in the Au ring top electrode region for further impedance characterization, which corresponds to yeast concentration c_y_ from c_y_ = 3.3 × 10^7^ cells/mL up to c_y_ =17 × 10^7^ cells/mL. The differences between dissimilar solvent i.e., DI water (Figure 2a–d) and glucose 10% (Figure 2e–h) in the optical microscopic images are not distinguishable. However, in the following sections, it will be demonstrated that the proposed biochips can be used for detecting the cell concentration and to distinguish between DI water and glucose as the solvent.

### 2.2. Modeling

**Figure 3:**
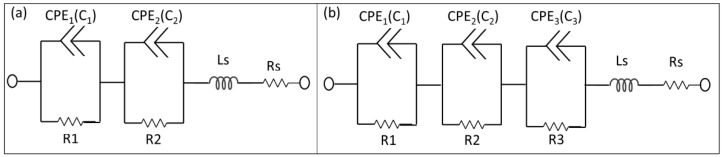
Electrical equivalent circuits used to model impedance spectra of the biochips (**a**) before (two pairs of constant phase elements (CPEs) and resistors) and (**b**) after inserting analyte into the ring electrode (3 pairs of CPEs and resistor). The equivalent circuit parameters Ls and Rs represent the interface properties of the circuit wiring.

Useful information can be obtained on the physicochemical properties of the system by measuring the small ac impedance signal of the biochip with medium without yeast cells and with medium with yeast cells, while using Impedance spectroscopy (ImS) [12]. ImS helps to observe the adhesion of biomaterials, because the adhesion changes the electrical behavior of the biochips and the electrical equivalent circuit is obtainable based on the electrical properties from the recorded Nyquist plots of the biochips and biochips with inserted biomaterial i.e., *S. cerevisiae* [13]. Few elements of the equivalent circuit model can be directly derived from the Nyquist curve in the frequency domain [14]. For example, a perfect semicircle in Nyquist curve describes a capacitor and a constant phase element (CPE) describes an imperfect semicircle in Nyquist curve [15]. From the other side, in a physical structure, the capacitance and resistance are associated with space charge polarization regions and with particular adsorption at the electrode and most of the structures with electrodes, normally contain a geometrical capacitance and a bulk resistance in parallel to it [16]. In the proposed p-n junction-based Si biochips, the bulk capacitance of the depletion region of the semiconductor and the capacitance of the Schottky contacts between the electrodes and semiconductor contribute to the impedance spectra of the biochips. We have used the complex nonlinear least square (CNLS) software to model and extract the equivalent circuit parameters from the electrical equivalent circuit. The Nyquist plots of the biochips reveal two imperfect semicircles. Two CPEs are used to model these two imperfect semicircles with the center below the x-axis in Nyquist plot (Figure 3a) [17].

CPE impedance is calculated as Z = 1/(Q_0_ (jω)n), where Q_0_ has the numerical value of admittance at ω = 1 rad/s with the unit S. The phase angle of the CPE impedance is frequency independent and it has a constant value of −(90 n) degrees and n is natural numbers. The initial values for the modeling parameters in CNLS software manually entered and the final values of the modeling parameters have been iteratively determined until the measured Nyquist plots is perfectly fit with the Nyquist plot generated by modeled values. In the final and perfect fit modeling values for the CPE component, the parameters RDE (resistance), TDE (relaxation time), and PDE (phase) can be obtained. The resistance part of CPE is determined by RDE, and the capacitive part Cp in CPE can be computed as Cp = (Q0 × RDE) (1/n)/RDE, where Ω_max_ is the frequency at which −Im{Z} is the maximum on Nyquist plot and Q0 = (TDE) × (PDE)/RDE. The values of the series resistor Rs and series Inductor Ls are achievable from the output modeling result as interface properties. The electrical equivalent circuit model of the biochips with no analyte consist of two pairs of CPEs that are in parallel with resistors (Figure 3a), while the electrical equivalent circuit of the biochips (with medium and high sensitivity) after adding suspension into the Au top electrode region consists of three pairs of CPEs and resistors (Figure 3b). The equivalent circuit parameters Rs and Ls contribute to the lead impedances. Note that the same equivalent circuit model has been applied to model the impedance change of biochips B5 and P5 after adding 1, 2, 3, 4, and 5 μL DI water (Figure 3a) or bacteria suspension (Figure 3b), i.e., *Lysinibacillus sphaericus* JG-A12 with OD_600_ = 4–16, in DI water, to 20 μL DI water.

## 3. Results

**Figure 4:**
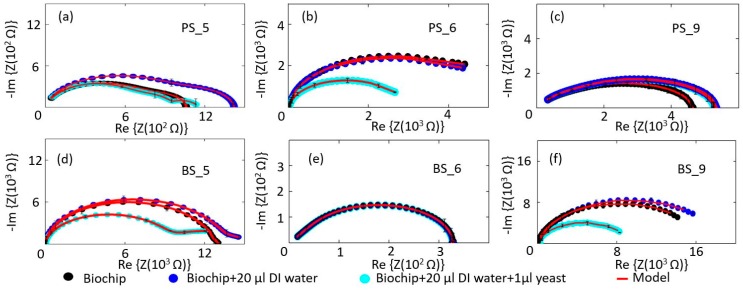
Measurement and modeling results for the (**a**–**c**) phosphor-implanted biochips, biochips with 20 μL DI water, biochip with 20 μL *S. cerevisiae* and (**d**–**f**) boron-implanted biochips, biochips with 20 μL DI water, biochip with 20 μL *S. cerevisiae*. Sensitivity level of the biochip to medium and to yeast *S. cerevisiae* has been summarized in Table 2. Biochips BS5 and PS5 show strong sensitivity to both medium and yeast.

Six different types of the biochips (Figure 1a–f) have been tested to evaluate the sensitivity level of each biochip to the DI water and to the *S. cerevisiae*. In the first step, the Nyquist plots for these six biochips have been measured and modeled. The black circular-dots in Figure 4 represent experimental data of the impedance of the biochip. In the second step, 20 μL DI water was inserted to the ring top electrode. Finally, 1 μL *S. cerevisiae* was added in to the ring top electrodes. The impedance characteristics of the unannealed biochips with low dopant concentrations, i.e., BS5 and PS5 (Figure 2a,d), have strong response to the DI water and to the *S. cerevisiae,* as illustrated in Figure 4a,d. For these two biochips, Nyquist curves due to adding DI water and adding *S. cerevisiae* are conveniently distinguishable. The annealed low phosphor-doped biochip PS6 and unannealed highly boron-doped biochip BS9 (Figure 2b,f), however do not have significant response to adding DI water. These two biochips show serious react to the adding *S. cerevisiae* though. In the case of adding *S. cerevisiae*, compare to these two biochips (PS6 and BS9), biochips BS5 and PS5 still have more remarkable response because in their Nyquist curves after adding yeast, additional semicircles can be easily observed.

The response of the unannealed highly phosphor-doped biochip PS9 (Figure 2c) to DI water and the *S. cerevisiae* is not noticeable and the biochip BS6 shows no react, neither to DI water nor to *S. cerevisiae*. Table 2 classifies the sensitivity of the biochips to the medium and to the *S. cerevisiae*.

**Figure 5:**
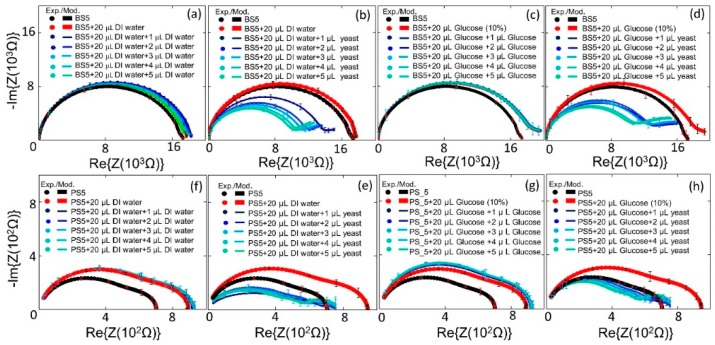
Experimental and modeled Nyquist plots of the Biochip BS5 with no filling and with DI water (20 µL) and (**a**) with additional DI water with volume from 1 μL to 5 μL, and (**b**) with additional *S. cerevisiae* volume from 1 μL to 5 μL. (**c**) no filling and with 10% glucose (20 µL) and with additional 10% glucose with volume from 1 μL to 5 μL, and (**d**) with additional *S. cerevisiae* volume from 1 μL to 5 μL. (**f**) biochip PS5 with no filling and with DI water (20 µL) and (**a**) with additional DI water with volume from 1 μL to 5 μL, and (**e**) with additional *S. cerevisiae* volume from 1 μL to 5 μL. (**g**) no filling and with 10% glucose (20 µL) and with additional 10% glucose with volume from 1–5 μL, and (**h**) with additional *S. cerevisiae* volume from 1–5 μL.

Based on the sensitivity (Table 2), biochips with strong sensitivity level to both medium and yeast have been selected for more detailed analysis. The impedance characteristics of biochips PS5 and BS5 are studied under the same experimental conditions. The ImS on biochips are measured without adding any analyte in the ring top electrode region (black thick curves in Figure 5), then, the ImS on biochips are recorded after adding 20 µL DI water (red thick curves in Figure 5a,b), and 20 µL glucose (red thick curves in Figure 5c,d) as medium. In the next step, for both biochips PS5 and BS5, the additional 1–5 µL *S. cerevisiae* suspension have been inserted into the ring top electrode region. Each measurement is repeated on individual biochip three times, and Figure 5 shows the corresponding experimental (circular dots) and modeled results (solid lines) with error bars. Impedance characteristics of the biochips with different medium (e.g., Figure 4a,c) indicate that, due to the obvious different curves, biochips are capable of sensing the different medium, which is not possible to be done with other techniques, e.g., with the optical microscope. As illustrated in Figure 2, no difference is visible between the microscopic image of the biochip with liquid (DI water) in Figure 2a–d and with medium (glucose) in Figure 2e–h. However, the proposed biochips BS5 and PS5 can sense these two different environmental categories and this would be one of the impressive aspects of these novel biochips. Along with this specification, the p-n junction-based biochips recognize the biomaterial in an exquisite form due to the significant changes in Nyquist curves.

**Figure A1:**
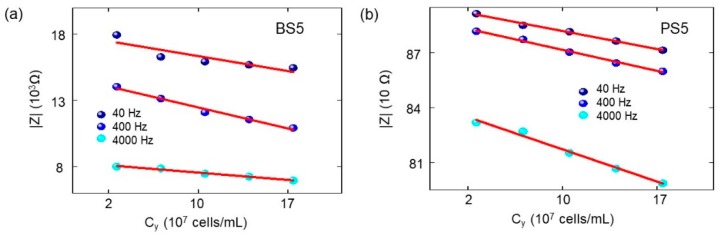
Impedance measured at a test frequency of 40 Hz, 400 Hz and 4000 Hz of biochip (**a**) BS5 (Table A16) and of biochip (**b**) PS5 (Table A17) in dependence on the yeast concentration c_y_ in DI water.

Significant sensitivity of the biochips BS5 and PS5 can be concluded based on observed changes in the impedance characteristics of the biochips with DI water or glucose (Figure 5a,c,f,g) and the corresponding impedance characteristics of the biochips with *S. cerevisiae* (Figure 5b,d,e,h). In these biochips, after adding the *S. cerevisiae* not only the resistive and capacitive impedance change, but also the additional semicircles in impedance characteristic of the biochips appear. At a fixed test frequency the impedance of the impedance biochips changes linearly in dependence on the yeast concentration (Figure A1 in Appendix B). However, the variation of impedance is smaller than 10% for c_y_, ranging from c_y_ = 3.3 × 10^7^ to c_y_ = 17 × 10^7^ cells/mL. Therefore, we modelled the impedance data in the whole test frequency range and analyzed up to six impedance model parameters in dependence on the yeast concentration. This has two advantages that are linked to the larger sensitivity of model parameters on the yeast cell concentration and that are linked to the larger number of independent parameters, revealing a linear dependence on the yeast concentration. It is noteworthy that the resistance of the boron-implanted biochip BS5 (black curve in plot Figure 5a) is generally larger than that of the phosphor-implanted biochip PS5 (black curve in plot Figure 5e). This is due to the lower conductivity of the p-type semiconductor, in which the holes are majority carriers in comparison to the n-type semiconductor, where the electrons are the majority carriers. The conductivity in these material is defined as σ = p.e.µh + n.e.µe, where e is the elemental charge, and the mobility of holes µh and the mobilities of electrons µe are 505 and 1450 cm^2^/Vs, respectively, and p represents the hole concentration and the n represents electron concentration.

**Figure 6:**
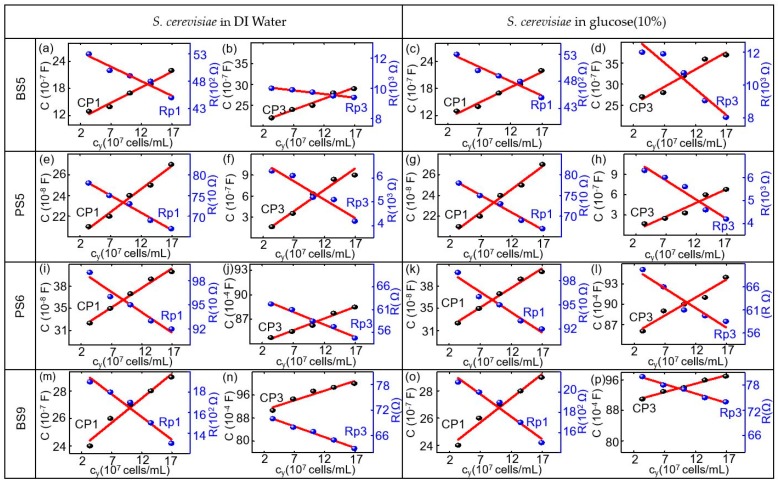
Modeled equivalent circuit parameters (dots) and linear fitting curve (red lines) for (**a**,**e**,**i**,**m**) Rp1, and Cp1 and for (**b**,**f**,**j**,**n**) Rp3 and CP3 of the biochip BS5,PS5,PS6,BS9 in dependence on the concentration yeast c_y_ in units of 10^7^ cells/mL in DI water and for (**c**,**g**,**k**,**o**) Rp1 and Cp1 and for (**d**,**h**,**l**,**p**) Rp3 and CP3 of the biochip BS5, PS5, PS6, BS9 in dependence on the concentration yeast c_y_ in units of 10^7^ cells/mL in the medium with 10% glucose.

The experimental impedance characteristics of all six biochips can be modeled by the equivalent circuit parameters in the equivalent circuit, as shown in Figure 3, which consists of two imperfect capacitors or CPEs (Cp1, Cp2), two parallel resistors (Rp1, Rp2), a contact resistor (Rs), and a contact inductor (Ls). ImS modeling parameters of these biochips are shown in Appendix A, Table A1, Table A2, Table A3, Table A4, Table A5, Table A6, Table A7, Table A8, Table A9, Table A10, Table A11, Table A12, Table A13, Table A14 and Table A15. It will be recalled that the equivalent circuits of the impedance spectra of the biochips with and without yeast are different (exceptional with biochip BS6 with no changes) due to the additional appeared semicircles. Accordingly, the composition and cell numbers of added *S. cerevisiae* to the Au top ring electrode region of the biochips can be determined from the modelled equivalent circuit parameters based on the experimental impedance characteristics of the biochips after adding yeast, as shown in Figure 3b. The parameters are three imperfect capacitors (Cp1, Cp2, Cp3) and three resistors (Rp1, Rp2, Rp3), a contact resistor (Rs), and a contact inductor (Ls). For calibration, the relation between the modeled equivalent circuit elements Rp1 (parallel resistor in 1st pair of the RC in modeling circuit in Ohm), Rp2 (parallel resistor in 2nd pair of the RC in modeling circuit in Ohm), Cp1 (parallel capacitor in 1st pair of the RC in modeling circuit in Farad), Cp2 (parallel capacitor in 2nd pair of the RC in modeling circuit in Farad), and Rp3 parallel resistor in 3rd pair of the RC in modeling circuit Ohm), and Cp3 (parallel capacitor in 2nd pair of the RC in modeling circuit in Farad) from impedance measurements and the nominal number of *S. cerevisiae* cells from optical microscopy measurements was determined. Appendix A, Table A1, Table A2, Table A3, Table A4, Table A5, Table A6, Table A7, Table A8, Table A9, Table A10, Table A11, Table A12, Table A13, Table A14 and Table A15, and Figure 6 show the corresponding ImS modeling results of the biochips. With the help of optical microscopic images, the ImS data and cell concentration observed with the optical density at 600 nm (OD_600_) has been calibrated. The impedance biochips have been calibrated by determining the modelled equivalent circuit elements of the impedance data measured on the biochips BS5, PS5, PS6, and BS9 in dependence on the concentration of yeast cells.

The calibration of the biochip is achieved in the volume range from 0–5 µL *S. cerevisiae* suspension in 20 µL DI water. OD_600_ of four corresponds to 3.3 × 10^7^ cells on the biochip if *S. cerevisiae* with 1 µL of concentration is applied to 20 µL DI water. For calibration, the dependency of the modeled equivalent circuit elements Rp1, Rp2, Cp1, Cp2, and Rp3 and Cp3 (from impedance modeling) on the nominal number of *S. cerevisiae* cells (from optical microscopy) was evaluated on the basis of the biochips. As demonstrated in Figure 6, equivalent circuit parameters Rp1, Cp1, Rp3, and Cp3 for biochips BS5, PS5, PS6, and BS9 have been proved to possess the linear dependence with the number of *S. cerevisiae* cells.

The values for the Cp1 and Rp1 for the biochips with *S. cerevisiae* in DI water are equal to the CP1 and Rp1 of the biochips with *S. cerevisiae* in glucose. This is due to the fact that Cp1 and Rp1 are described by the Schottky contact parameters of the biochips. Figure 6 shows the modeling parameters for the two out of six impedance biochips BS5 and PS6, which are strongly sensitive to the addition of both medium without yeast and medium with yeast. The remarkable point is that the equivalent circuit parameters can be linearly fit and, as it can be concluded from the plots and numerical values in Table A1, Table A2, Table A3, Table A4, Table A5, Table A6, Table A7, Table A8, Table A9, Table A10, Table A11, Table A12, Table A13, Table A14 and Table A15, for the biochip BS5 and PS6 with high sensitivity level, the resistive (Rp3) and capacitive (Cp3) response of the biochip with *S. cerevisiae* in glucose is even larger than that of *S. cerevisiae* in DI. For the biochips PS5, the capacitive (Cp3) changes are remarkable with different medium and, for biochip BS9, the resistive impedance (Rp3) can be taken as the valuable scale for changing the impedance for different medium. As demonstrated in Figure 6, equivalent circuit parameters Rp1, Cp1, Rp3, and Cp3 for biochips BS5, PS5, PS6, and BS9 linearly depend on the number of *S. cerevisiae*. The linear relationship with the nominal number of *S. cerevisiae* cells for the biochips is in the range from 3.3 × 10^7^ cells/mL to 17 × 10^7^ cells/mL. The modeling parameters Rp1 and Cp1 represent the Schottky contact at the electrode/semiconductor interface. If the size of contact area is denoted as A, by adding *S. cerevisiae* suspension to the top electrode region of biochips, the area of the top contact is increased. According to equation Rp1 = ρ(d/A), where d denotes the thickness of the Schottky barrier, the resistance is reversely related to the area A. Thus, there is a reduction in resistance Rp1 by adding the yeast suspension. If we consider Cp1 = ε(A/d) with ε as the permittivity of semiconductor, the relationship between Cp1 and A results in the increasing Cp1 with increasing *S. cerevisiae* suspension. The Rp2 and Cp2 correspond to the impedance of semiconductors Si:B in phosphor-implanted and Si:P in boron-implanted biochips and the Rp3 and Cp3 pair represents the impedance of *S. cerevisiae* suspension, which is added into the Au top electrode region. In brief, the linear impedance variation depends on the *S. cerevisiae* concentration for the Si biochips and has been modeled with four parameters Rp1, Cp1, Rp3, and CP3. Therefore, a multiparameter determination of the *S. cerevisiae* concentration can be performed continuous detection while using the Si biochips.

## 4. Discussion

**Figure 7:**
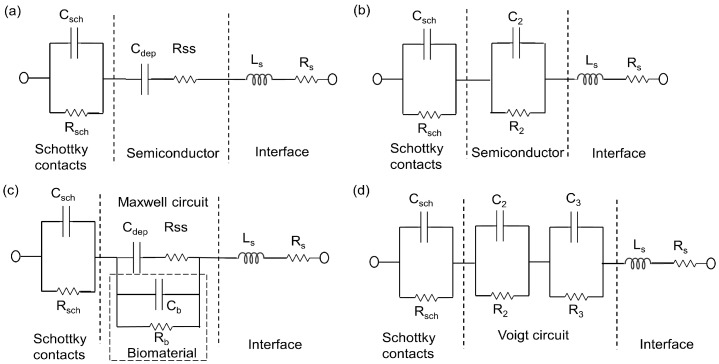
Equivalent circuit models of biochip without analyte (**a**) based on the physiochemical structure of biochip and (**b**) based on the associated RC pairs. The parallel capacitor C_2_ and resistor R_2_ are transferred from C_dep_ and Rss. Equivalent circuit models of biochip with analyte (**c**) in Maxwell fashion and in (**d**) Voigt fashion. The Voigt fashion can be transferred into Maxwell fashion while using Equations (1)–(3).

The surface charge of yeasts cell membrane is negative due to the presence of carboxyl, phosphoryl, and hydroxyl groups [18]. The phosphorylation of mannosyl side chains belonging to the mannoproteins of yeasts cell wall is responsible for the anionic (negative) surface charge [19]. In this work we analyzed the interaction between the surface of inner ring region of the top electrode of the impedance biochips, i.e., a thin silica layer on Si P-N junction, and the yeast cells. First, we analyzed the impedance data of the biochip without medium in the inner region of the ring top electrode and we then analyzed the impedance of the biochip after inserting medium without yeast cells and with different number of yeast cells. The added yeast cells with negative surface charge are expected to be attached to the surface of the impedance biochip, thus reducing the contact resistance of the inner ring region of the impedance chip. The pair of resistor (R_b_) and capacitor (C_b_) account for the change of the contact of the inner ring region (Figure 7c). R_b_ and C_b_ have been modeled (Figure A1). We assume that the pH value of the medium is not changed by adding more (1, 2, 3, 4, and 5 mL medium with yeast cells) yeast cells to the inner ring region of the impedance chip, i.e., during changing the concentration of yeast cells from 3.3 × 10^7^ to c_y_ = 17.0 × 10^7^ cells/mL, i.e., by a factor of 20, because the formation of pH decreasing CO_2_ from glucose consumption during cultivation will only occur if there is no buffering agent within the cultivation medium. It would be directly estimated that the associated resistor (R) and capacitor (C) can be used for describing the physical structures of the biochips by analyzing the Nyquist plots of the biochips BS5 and PS5 with strong sensitivity level. As these Nyquist plots suggest, pairs of resistor and capacitor (RC) are needed in the electrical equivalent circuit due to the existence of two non-overlapping semicircles [20]. This semicircle is caused by the Schottky contacts, which formed at the interfaces between Au top and bottom electrode/semiconductor. These two metal/semiconductor Schottky contacts can be represented by one pair of CPE and resistor in the electrical equivalent circuit [21]. The biochips also contain another basic part, which is the p-n junction. The p-n junction is a boundary or interface between p- and n-type silicon. A depletion region is formed in the interface of these two semiconductor types and it consists of depletion capacitor (C_dep_) and semiconductor resistor (R_ss_). Thus, the biochip can be described by the introduced model in Figure 7a, with two capacitors three resistors, and one inductor. This model that is based on the physical mechanism of the biochip can then be transferred to proper model of electrical equivalent circuit shown in the Figure 7b by converting the series structure of the C_dep_ and Rss to the parallel C_2_//R_2_ (Figure 7b), where C_2_ = C_dep_·(Q^2^/(1 + Q^2^)), R_2_ = R_ss_·(1 + Q^2^) with the definition of Q = 1/(ω·C_dep_·Rss) [22]. Thus, the impedance spectra of biochips PS5 and BS5, as depicted in thick black curves in Figure 4, which can be modeled by using equivalent circuits in Figure 7b, based on the transferring the physiochemical model to electrical equivalent circuit.

Since the DI water and glucose 10% with yeast applied to the ring top electrode, a two-phase electrode contact [23] have been developed, where one phase is resulted by the electrodes of biochip and the other phase is related to the added analyte [24]. The consequence of the additional phase is the appearance of an additional semicircle in the corresponding Nyquist plot and the impedance magnitude of the biochips after adding analyte is directly related to cell concentration. Based on the measurement result of the overall impedance (Figure A1, Table A16 and Table A17), the magnitude at different frequencies is decreasing with increasing *S. cerevisiae* concentration, which indicates the validity of parallel connection of R_b_C_b_ pairs in the physiochemical model of the biochip with the biomaterial in Figure 7c. In electrical equivalent circuit with inserted *S. cerevisiae* (Figure 7d), an additional R_3_C_3_ pair is used for modeling. This electrical modeling circuit needs to be equal to the physiochemical model.

(1) $$C_{2,3}=2\;C_{b}{\left(1\mp \frac{\frac{Rss}{Rb}-\frac{Cb}{Cdep+1}}{k^{1/2}}\right)}^{-1}$$

(2) $$R_{2,3}=\frac{Rb}{2}\left(1\pm \frac{\frac{Cb}{Cdep}-\frac{Rss}{Rb+1}}{k^{1/2}}\right)$$

(3) $$k={\left(\frac{Cb}{Cdep}+\frac{Rss}{Rb}+1\right)}^{2}-\frac{4Cb.Rss}{Cdep.Rb}$$

To transfer these two models, we will employ two equivalent circuits that are equal at any frequencies. Maxwell circuit in Figure 7c can be transferred into Voigt circuit, as illustrated in Figure 7d by utilizing the Equations (1)–(3) [25].

## 5. Summary and Outlook

We reported on the disturbing-free determination of yeast (*S. cerevisiae*) concentration with an optical density of OD_600_ = 4–16 in DI water and in glucose while using six different types of Si p-n and Si n-p junction based impedance biochips, which have been prepared by different ion implantation conditions into n-Si and p-Si wafers, respectively. Accompanied by two out of the six biochips, monitoring the yeast during the cultivation with considerable high precision and considerable time efficiency is attainable. The impedance characteristics of the impedance biochips are discussed with a focus on changes of the impedance spectra before and after adding the yeast suspension *S. cerevisiae* in the inner region of the top electrode of the biochips. We developed an equivalent circuit model for the impedance biochip with a two-phase electrode with four modeling parameters being very sensitive to impedance changes of the inner region of the top electrode. Those parameters, i.e., Rp1, Cp1, Rp3, and Cp3, reveal a linear dependence on the yeast concentration for the biochips with strong and medium sensitivity level. Such a linear dependence enables the quantitative determination of the yeast concentration. The sensitivity of the impedance biochips is the largest for two out of the six different types of biochips and optimizing the geometry of the top electrode can further increase it. Next, we will study differences in impedance data recorded on the biochips, where live and dead yeast cells with same optical density are added in the inner region of the top electrode.

## Figures and Tables

**Table 1:** Implantation parameters and annealing conditions of phosphor implanted (phosphorous into Si:B) and boron implanted (boron into Si:P) biochips. The Au ring top electrodes and unstructured Au bottom contacts have been prepared after ion implantation. Different ion species together with ion energy, ion fluence, and annealing conditions have been applied when preparing the six different types of impedance biochips.

Biochip	Implanted Ion	Ion Energy (MeV)	Ion Fluence (cm^−2^)	Annealing
PS5	Phosphorous	1.00	3 × 10^13^	No
BS5	Boron	0.45	3 × 10^13^	No
PS6	Phosphorous	1.00	3 × 10^13^	Yes, at 900 °C
BS6	Boron	0.45	3 × 10^13^	Yes, at 900 °C
PS9	Phosphorous	1.00	3 × 10^15^	No
BS9	Boron	0.40	8 × 10^15^	No

**Table 2:** Sensitivity level for the manufactured biochips to DI water and to yeast. 20 µL DI water and 1 µL *S. cerevisiae* inserted to the ring top electrode for testing the sensitivity of the biochips to medium and to yeast, respectively.

Bio-Chip ID	Sensitivity to Medium	Sensitivity to Yeast
PS5	Strong	Strong
BS5	Strong	Strong
PS6	Medium	Strong
BS6	Weak	Weak
PS9	Strong	Weak
BS9	Medium	Strong

## Appendix A

**Table A1:** Modeled impedance parameters Cp1, Rp1, Cp2, Rp2, Cp3, Rp3, Rs, and Ls of biochip BS5, with DI water.

Circuit Element	Cp1 (F)	Rp1 (Ω)	Cp2 (F)	Rp2 (Ω)	Cp3 (F)	Rp3 (Ω)	Rs (Ω)	Ls (H)
BS5	4.6 × 10^−9^	1.2 × 10^4^	7.5 × 10^−15^	9.4 × 10^6^	-	-	2.1 × 10^−9^	2.4 × 10^−15^
BS5 + W20	4.6 × 10^−9^	1.5 × 10^4^	7.5 × 10^−15^	9.4 × 10^6^	2.6 × 10^−6^	6.2 × 10^5^	2.1 × 10^−9^	2.4 × 10^−15^
BS5 + W20 + W1	4.5 × 10^−9^	1.5 × 10^4^	7.5 × 10^−15^	9.4 × 10^6^	2.9 × 10^−6^	2.1 × 10^3^	2.1 × 10^−9^	2.4 × 10^−15^
BS5 + W20 + W2	4.7 × 10^−9^	1.4 × 10^4^	7.5 × 10^−15^	9.4 × 10^6^	3.5 × 10^−6^	2.3 × 10^3^	2.1 × 10^−9^	2.4 × 10^−15^
BS5 + W20 + W3	4.8 × 10^−9^	1.3 × 10^4^	7.5 × 10^−15^	9.4 × 10^6^	3.9 × 10^−6^	2.5 × 10^3^	2.1 × 10^−9^	2.4 × 10^−15^
BS5 + W20 + W4	4.9 × 10^−9^	1.2 × 10^4^	7.5 × 10^−15^	9.4 × 10^6^	4.2 × 10^−6^	4.3 × 10^4^	2.1 × 10^−9^	2.4 × 10^−15^
BS5 + W20 + W5	5.0 × 10^−9^	1.1 × 10^4^	7.5 × 10^−15^	9.4 × 10^6^	4.5 × 10^−6^	5.0 × 10^3^	2.1 × 10^−9^	2.4 × 10^−15^

W20 = 20 µL DI water, W1 = 1 µL DI Water, W2 = 2 µL DI Water, W3 = 3 µL DI Water, W4 = 4 µL DI Water, W5 = 5 µL DI Water.

**Table A2:** Modeled impedance parameters Cp1, Rp1, Cp2, Rp2, Cp3, Rp3, Rs, and Ls of biochip BS5, with DI water and *S. cerevisiae.*

Circuit Element	Cp1 (F)	Rp1 (Ω)	Cp2 (F)	Rp2 (Ω)	Cp3 (F)	Rp3 (Ω)	Rs (Ω)	Ls(H)
BS5	4.6 × 10^−9^	1.2 × 10^4^	7.5 × 10^−15^	9.4 × 10^6^	-	-	2.1 × 10^−9^	2.4 × 10^−15^
BS5 + W20	4.6 × 10^−9^	1.5 × 10^4^	7.5 × 10^−15^	9.4 × 10^6^	2.6 × 10^−6^	6.2 × 10^2^	2.1 × 10^−9^	2.4 × 10^−15^
BS5 + W20 + Y1	1.3 × 10^−8^	5.3 × 10^3^	7.5 × 10^−15^	9.4 × 10^6^	2.2 × 10^−7^	1.0 × 10^4^	2.1 × 10^−9^	2.4 × 10^−15^
BS5 + W20 + Y2	1.4 × 10^−8^	5.0 × 10^3^	7.5 × 10^−15^	9.4 × 10^6^	2.4 × 10^−7^	9.9 × 10^3^	2.1 × 10^−9^	2.4 × 10^−15^
BS5 + W20 + Y3	1.7 × 10^−8^	4.9 × 10^3^	7.5 × 10^−15^	9.4 × 10^6^	2.5 × 10^−7^	9.7 × 10^3^	2.1 × 10^−9^	2.4 × 10^−15^
BS5 + W20 + Y4	1.9 × 10^−8^	4.8 × 10^3^	7.5 × 10^−15^	9.4 × 10^6^	2.8 × 10^−7^	9.5 × 10^3^	2.1 × 10^−9^	2.4 × 10^−15^
BS5 + W20 + Y5	2.2 × 10^−8^	4.5 × 10^3^	7.5 × 10^−15^	9.4 × 10^6^	2.9 × 10^−7^	9.4 × 10^3^	2.1 × 10^−9^	2.4 × 10^−15^

W20 = 20 µL DI water, Y1 = 1 µL yeast *Saccharomyces*, Y2 = 2 µL yeast *Saccharomyces*, Y3 = 3 µL yeast *Saccharomyces*, Y4 = 4 µL yeast *Saccharomyces*, Y5 = 5 µL yeast *Saccharomyces*.

**Table A3:** Modeled impedance parameters Cp1, Rp1, Cp2, Rp2, Cp3, Rp3, Rs, and Ls of biochip BS5, with 10% glucose.

Circuit Element	Cp1 (F)	Rp1 (Ω)	Cp2 (F)	Rp2 (Ω)	Cp3 (F)	Rp3 (Ω)	Rs (Ω)	Ls (H)
BS5	4.6 × 10^−9^	1.2 × 10^4^	7.5 × 10^−15^	9.4 × 10^6^	-	-	2.1 × 10^−9^	2.4 × 10^−15^
BS5 + G20	4.6 × 10^−9^	1.5 × 10^4^	7.5 × 10^−15^	9.4 × 10^6^	2.6 × 10^−6^	6.2 × 10^2^	2.1 × 10^−9^	2.4 × 10^−15^
BS5 + G20 + G1	4.3 × 10^−9^	1.4 × 10^4^	7.5 × 10^−15^	9.4 × 10^6^	2.9 × 10^−6^	5.0 × 10^2^	2.1 × 10^−9^	2.4 × 10^−15^
BS5 + G20 + G2	4.4 × 10^−9^	1.3 × 10^4^	7.5 × 10^−15^	9.4 × 10^6^	3.8 × 10^−6^	4.0 × 10^2^	2.1 × 10^−9^	2.4 × 10^−15^
BS5 + G20 + G3	4.5 × 10^−9^	1.1 × 10^4^	7.5 × 10^−15^	9.4 × 10^6^	4.1 × 10^−6^	2.2 × 10^2^	2.1 × 10^−9^	2.4 × 10^−15^
BS5 + G20 + G4	4.6 × 10^−9^	1.0 × 10^4^	7.5 × 10^−15^	9.4 × 10^6^	4.5 × 10^−6^	2.1 × 10^2^	2.1 × 10^−9^	2.4 × 10^−15^
BS5 + G20 + G5	4.7 × 10^−9^	0.9 × 10^4^	7.5 × 10^−15^	9.4 × 10^6^	4.9 × 10^−6^	1.9 × 10^2^	2.1 × 10^−9^	2.4 × 10^−15^

G20 = 20 µL 10% Glucose, G1 = 1 µL 10% Glucose, G2 = 2 µL 10% Glucose, G3 = 3 µL 10% Glucose, G4 = 4 µL 10% Glucose, G5 = 5 µL 10% Glucose.

**Table A4:** Modeled impedance parameters Cp1, Rp1, Cp2, Rp2, Cp3, Rp3, Rs, and Ls of biochip BS5, with 10% glucose as medium and *S. cerevisiae*.

Circuit Element	Cp1 (F)	Rp1 (Ω)	Cp2 (F)	Rp2 (Ω)	Cp3 (F)	Rp3 (Ω)	Rs (Ω)	Ls (H)
BS5	4.6 × 10^−9^	1.2 × 10^4^	7.5 × 10^−15^	9.4 × 10^6^	-	-	2.1 × 10^−9^	2.4 × 10^−15^
BS5 + W20	4.6 × 10^−9^	1.5 × 10^4^	7.5 × 10^−15^	9.4 × 10^6^	3.0 × 10^−6^	6.2 × 10^2^	2.1 × 10^−9^	2.4 × 10^−15^
BS5 + G20 + Y1	1.3 × 10^−8^	5.3 × 10^3^	7.5 × 10^−15^	9.4 × 10^6^	2.7 × 10^−6^	1.2 × 10^4^	2.1 × 10^−9^	2.4 × 10^−15^
BS5 + G20 + Y2	1.4 × 10^−8^	5.0 × 10^3^	7.5 × 10^−15^	9.4 × 10^6^	2.8 × 10^−6^	1.1 × 10^4^	2.1 × 10^−9^	2.4 × 10^−15^
BS5 + G20 + Y3	1.7 × 10^−8^	4.9 × 10^3^	7.5 × 10^−15^	9.4 × 10^6^	3.2 × 10^−6^	1.0 × 10^4^	2.1 × 10^−9^	2.4 × 10^−15^
BS5 + G20 + Y4	1.9 × 10^−8^	4.8 × 10^3^	7.5 × 10^−15^	9.4 × 10^6^	3.6 × 10^−6^	9.0 × 10^3^	2.1 × 10^−9^	2.4 × 10^−15^
BS5 + G20 + Y5	2.2 × 10^−8^	4.5 × 10^3^	7.5 × 10^−15^	9.4 × 10^6^	3.7 × 10^−6^	8.0 × 10^3^	2.1 × 10^−9^	2.4 × 10^−15^

G20 = 20 µL 10% Glucose, Y1 = 1 µL yeast *Saccharomyces*, Y2 = 2 µL yeast *Saccharomyces*, Y3 = 3 µL yeast *Saccharomyces*, Y4 = 4 µL yeast *Saccharomyces*, Y5 = 5 µL yeast *Saccharomyces*.

**Table A5:** Modeled impedance parameters Cp1, Rp1, Cp2, Rp2, Cp3, Rp3, Rs, and Ls of biochip PS5, with added DI water.

Circuit Element	Cp1 (F)	Rp1 (Ω)	Cp2 (F)	Rp2 (Ω)	Cp3 (Ω)	Rp3 (Ω)	Rs (Ω)	Ls (H)
PS5	8.5 × 10^−9^	6.5 × 10^2^	5.1 × 10^−15^	9.4 × 10^6^	-	-	3.6 × 10^−8^	2.4 × 10^−15^
PS5 + W20	8.1 × 10^−9^	8.1 × 10^2^	5.1 × 10^−15^	9.4 × 10^6^	1.4 × 10^−7^	2.8 × 10^2^	3.6 × 10^−8^	2.4 × 10^−15^
PS5 + W20 + W1	7.8 × 10^−9^	8.7 × 10^2^	5.1 × 10^−15^	9.4 × 10^6^	1.6 × 10^−7^	3.7 × 10^2^	3.6 × 10^−8^	2.4 × 10^−15^
PS5 + W20 + W2	7.6 × 10^−9^	8.6 × 10^2^	5.1 × 10^−15^	9.4 × 10^6^	1.5 × 10^−7^	4.2 × 10^3^	3.6 × 10^−8^	2.4 × 10^−15^
PS5 + W20 + W3	7.5 × 10^−9^	8.4 × 10^2^	5.1 × 10^−15^	9.4 × 10^6^	1.6 × 10^−7^	4.3 × 10^3^	3.6 × 10^−8^	2.4 × 10^−15^
PS5 + W20 + W4	7.4 × 10^−9^	8.3 × 10^2^	5.1 × 10^−15^	9.4 × 10^6^	1.7 × 10^−7^	4.4 × 10^3^	3.6 × 10^−8^	2.4 × 10^−15^
PS5 + W20 + W5	7.3 × 10^−9^	8.1 × 10^2^	5.1 × 10^−15^	9.4 × 10^6^	1.8 × 10^−7^	4.5 × 10^3^	3.6 × 10^−8^	2.4 × 10^−15^

W20 = 20 µL DI water, W1 = 1 µL DI Water, W2 = 2 µL DI Water, W3 = 3 µL DI Water, W4 = 4 µL DI Water, W5 = 5 µL DI Water.

**Table A6:** Modeled impedance parameters Cp1, Rp1, Cp2, Rp2, Cp3, Rp3, Rs, and Ls of biochip PS5, with DI water and *S. cerevisiae*.

Circuit Element	Cp1 (F)	Rp1 (Ω)	Cp2 (F)	Rp2 (Ω)	Cp3 (Ω)	Rp3 (Ω)	Rs (Ω)	Ls (H)
PS5	8.5 × 10^−9^	6.5 × 10^2^	5.1 × 10^−15^	9.4 × 10^6^	-	-	3.6 × 10^−8^	2.4 × 10^−15^
PS5 + W20	8.1 × 10^−9^	8.1 × 10^2^	5.1 × 10^−15^	9.4 × 10^6^	1.4 × 10^−7^	2.8 × 10^2^	3.6 × 10^−8^	2.4 × 10^−15^
PS5 + W20 + Y1	2.1 × 10^−9^	7.8 × 10^2^	5.1 × 10^−15^	9.4 × 10^6^	1.6 × 10^−7^	6.3 × 10^3^	3.6 × 10^−8^	2.4 × 10^−15^
PS5 + W20 + Y2	2.2 × 10^−9^	7.5 × 10^2^	5.1 × 10^−15^	9.4 × 10^6^	3.5 × 10^−7^	6.1 × 10^3^	3.6 × 10^−8^	2.4 × 10^−15^
PS5 + W20 + Y3	2.4 × 10^−9^	7.3 × 10^2^	5.1 × 10^−15^	9.4 × 10^6^	6.3 × 10^−7^	5.2 × 10^3^	3.6 × 10^−8^	2.4 × 10^−15^
PS5 + W20 + Y4	2.5 × 10^−9^	6.9 × 10^2^	5.1 × 10^−15^	9.4 × 10^6^	8.4 × 10^−7^	5.1 × 10^3^	3.6 × 10^−8^	2.4 × 10^−15^
PS5 + W20 + Y5	2.7 × 10^−9^	6.7 × 10^2^	5.1 × 10^−15^	9.4 × 10^6^	9.0 × 10^−7^	4.2 × 10^3^	3.6 × 10^−8^	2.4 × 10^−15^

W20 = 20 µL DI water, Y1 = 1 µL yeast *Saccharomyces*, Y2 = 2 µL yeast *Saccharomyces*, Y3 = 3 µL yeast *Saccharomyces*, Y4 = 4 µL yeast *Saccharomyces*, Y5 = 5 µL yeast *Saccharomyces*.

**Table A7:** Modeled impedance parameters Cp1, Rp1, Cp2, Rp2, Cp3, Rp3, Rs, and Ls of biochip pS5, with 10% glucose.

Circuit Element	Cp1 (F)	Rp1 (Ω)	Cp2 (F)	Rp2 (Ω)	Cp3 (Ω)	Rp3 (Ω)	Rs (Ω)	Ls (H)
PS5	8.5 × 10^−9^	6.5 × 10^2^	5.1 × 10^−15^	9.4 × 10^6^	-	-	3.6 × 10^−8^	2.4 × 10^−15^
PS5 + G20	8.1 × 10^−9^	8.1 × 10^2^	5.1 × 10^−15^	9.4 × 10^6^	1.4 × 10^−6^	2.8 × 10^2^	3.6 × 10^−8^	2.4 × 10^−15^
PS5 + G20 + G1	8.1 × 10^−9^	9.1 × 10^2^	5.1 × 10^−15^	9.4 × 10^6^	2.1 × 10^−6^	3.7 × 10^2^	3.6 × 10^−8^	2.4 × 10^−15^
PS5 + G20 + G2	7.9 × 10^−9^	8.9 × 10^2^	5.1 × 10^−15^	9.4 × 10^6^	2.0 × 10^−6^	4.8 × 10^2^	3.6 × 10^−8^	2.4 × 10^−15^
PS5 + G20 + G3	7.8 × 10^−9^	8.8 × 10^2^	5.1 × 10^−15^	9.4 × 10^6^	1.8 × 10^−6^	4.5 × 10^2^	3.6 × 10^−8^	2.4 × 10^−15^
PS5 + G20 + G4	7.7 × 10^−9^	8.5 × 10^2^	5.1 × 10^−15^	9.4 × 10^6^	1.7 × 10^−6^	4.3 × 10^2^	3.6 × 10^−8^	2.4 × 10^−15^
PS5 + G20 + G5	7.6 × 10^−9^	8.3 × 10^2^	5.1 × 10^−15^	9.4 × 10^6^	1.6 × 10^−6^	4.1 × 10^2^	3.6 × 10^−8^	2.4 × 10^−15^

G20 = 20 µL 10% Glucose, G1 = 1 µL 10% Glucose, G2 = 2 µL 10% Glucose, G3 = 3 µL 10% Glucose, G4 = 4 µL 10% Glucose, G5 = 5 µL 10% Glucose.

**Table A8:** Modeled impedance parameters Cp1, Rp1, Cp2, Rp2, Cp3, Rp3, Rs, and Ls of biochip PS5, with 10% glucose as medium and *S. cerevisiae*.

Circuit Element	Cp1 (F)	Rp1 (Ω)	Cp2 (F)	Rp2 (Ω)	Cp3 (Ω)	Rp3 (Ω)	Rs (Ω)	Ls (H)
PS5	8.5 × 10^−9^	6.5 × 10^2^	5.1 × 10^−15^	9.4 × 10^6^	-	-	3.6 × 10^−8^	2.4 × 10^−15^
PS5 + G20	8.1 × 10^−9^	8.1 × 10^2^	5.1 × 10^−15^	9.4 × 10^6^	1.6 × 10^−7^	1.9 × 10^2^	3.6 × 10^−8^	2.4 × 10^−15^
PS5 + G20 + Y1	2.1 × 10^−9^	7.8 × 10^2^	5.1 × 10^−15^	9.4 × 10^6^	1.7 × 10^−7^	6.3 × 10^3^	3.6 × 10^−8^	2.4 × 10^−15^
PS5 + G20 + Y2	2.2 × 10^−9^	7.5 × 10^2^	5.1 × 10^−15^	9.4 × 10^6^	2.5 × 10^−7^	6.0 × 10^3^	3.6 × 10^−8^	2.4 × 10^−15^
PS5 + G20 + Y3	2.4 × 10^−9^	7.3 × 10^2^	5.1 × 10^−15^	9.4 × 10^6^	3.3 × 10^−7^	5.6 × 10^3^	3.6 × 10^−8^	2.4 × 10^−15^
PS5 + G20 + Y4	2.5 × 10^−9^	6.9 × 10^2^	5.1 × 10^−15^	9.4 × 10^6^	6.0 × 10^−7^	4.6 × 10^3^	3.6 × 10^−8^	2.4 × 10^−15^
PS5 + G20 + Y5	2.7 × 10^−9^	6.7 × 10^2^	5.1 × 10^−15^	9.4 × 10^6^	6.8 × 10^−7^	4.2 × 10^3^	3.6 × 10^−8^	2.4 × 10^−15^

G20 = 20 µL 10% Glucose, Y1 = 1 µL yeast *Saccharomyces*, Y2 = 2 µL yeast *Saccharomyces*, Y3 = 3 µL yeast *Saccharomyces,* Y4 = 4 µL yeast *Saccharomyces*, Y5 = 5 µL yeast *Saccharomyces*.

**Table A9:** Modeled impedance parameters Cp1, Rp1, Cp2, Rp2, Cp3, Rp3, Rs, and Ls of biochip BS6, with DI water and *S. cerevisiae*.

Circuit Element	Cp1 (F)	Rp1 (Ω)	Cp2 (F)	Rp2 (Ω)	Cp3(Ω)	Rp3 (Ω)	Rs (Ω)	Ls (H)
BS6	1.2 × 10^−8^	1.9 × 10^2^	1.3 × 10^−10^	1.5 × 10^2^	-	-	4.7 × 10^−8^	2.4 × 10^−15^
BS6 + varing analyte	1.2 × 10^−8^	1.9 × 10^2^	1.3 × 10^−10^	1.5 × 10^2^	-	-	4.7 × 10^−8^	2.4 × 10^−15^

**Table A10:** Modeled impedance parameters Cp1, Rp1, Cp2, Rp2, Cp3, Rp3, Rs, and Ls of biochip PS6, with DI water and *S. cerevisiae*.

Circuit Element	Cp1 (F)	Rp1 (Ω)	Cp2 (F)	Rp2 (Ω)	Cp3 (Ω)	Rp3 (Ω)	Rs (Ω)	Ls (H)
PS6	6.2 × 10^−9^	1.7 × 10^3^	8.1 × 10^−9^	6.3 × 10^2^	-	-	2.7 × 10^−8^	2.4 × 10^−15^
PS6 + W20	7.2 × 10^−9^	2.9 × 10^3^	8.1 × 10^−9^	6.3 × 10^2^	7.2 × 10^−5^	2.6 × 10^1^	2.7 × 10^−8^	2.4 × 10^−15^
PS6 + W20 + Y1	3.4 × 10^−9^	9.9 × 10^2^	8.1 × 10^−9^	6.3 × 10^2^	8.4 × 10^−5^	6.1 × 10^1^	2.7 × 10^−8^	2.4 × 10^−15^
PS6 + W20 + Y2	3.6 × 10^−9^	9.6 × 10^2^	8.1 × 10^−9^	6.3 × 10^2^	8.5 × 10^−5^	6.0 × 10^1^	2.7 × 10^−8^	2.4 × 10^−15^
PS6 + W20 + Y3	3.8 × 10^−9^	9.5 × 10^2^	8.1 × 10^−9^	6.3 × 10^2^	8.6 × 10^−5^	5.8 × 10^1^	2.7 × 10^−8^	2.4 × 10^−15^
PS6 + W20 + Y4	4.0 × 10^−9^	9.3 × 10^2^	8.1 × 10^−9^	6.3 × 10^2^	8.8 × 10^−5^	5.7 × 10^1^	2.7 × 10^−8^	2.4 × 10^−15^
PS6 + W20 + Y5	4.1 × 10^−9^	9.2 × 10^2^	8.1 × 10^−9^	6.3 × 10^2^	8.9 × 10^−5^	5.5 × 10^1^	2.7 × 10^−8^	2.4 × 10^−15^

W20 = 20 µL DI water, Y1 = 1 µL yeast *Saccharomyces*, Y2 = 2 µL yeast *Saccharomyces*, Y3 = 3 µL yeast *Saccharomyces*, Y4 = 4 µL yeast *Saccharomyces*, Y5 = 5 µL yeast *Saccharomyces*.

**Table A11:** Modeled impedance parameters Cp1, Rp1, Cp2, Rp2, Cp3, Rp3, Rs, and Ls of biochip PS6, with 10% glucose as medium and *S. cerevisiae*.

Circuit Element	Cp1 (F)	Rp1 (Ω)	Cp2 (F)	Rp2 (Ω)	Cp3 (Ω)	Rp3 (Ω)	Rs (Ω)	Ls (H)
PS6	6.2 × 10^−9^	1.7 × 10^3^	8.1 × 10^−9^	6.3 × 10^2^	-	-	2.7 × 10^−8^	2.4 × 10^−15^
PS6 + G20	7.4 × 10^−9^	3.1 × 10^3^	8.1 × 10^−9^	6.3 × 10^2^	7.5 × 10^−5^	2.9 × 10^1^	2.7 × 10^−8^	2.4 × 10^−15^
PS6 + G20 + Y1	3.4 × 10^−9^	9.9 × 10^2^	8.1 × 10^−9^	6.3 × 10^2^	8.6 × 10^−5^	6.7 × 10^1^	2.7 × 10^−8^	2.4 × 10^−15^
PS6 + G20 + Y2	3.6 × 10^−9^	9.6 × 10^2^	8.1 × 10^−9^	6.3 × 10^2^	8.9 × 10^−5^	6.4 × 10^1^	2.7 × 10^−8^	2.4 × 10^−15^
PS6 + G20 + Y3	3.8 × 10^−9^	9.5 × 10^2^	8.1 × 10^−9^	6.3 × 10^2^	9.0 × 10^−5^	6.0 × 10^1^	2.7 × 10^−8^	2.4 × 10^−15^
PS6 + G20 + Y4	4.0 × 10^−9^	9.3 × 10^2^	8.1 × 10^−9^	6.3 × 10^2^	9.1 × 10^−5^	5.9 × 10^1^	2.7 × 10^−8^	2.4 × 10^−15^
PS6 + G20 + Y5	4.1 × 10^−9^	9.2 × 10^2^	8.1 × 10^−9^	6.3 × 10^2^	9.4 × 10^−5^	5.8 × 10^1^	2.7 × 10^−8^	2.4 × 10^−15^

G20 = 20 µL 10% Glucose, Y1 = 1 µL yeast *Saccharomyces*, Y2 = 2 µL yeast *Saccharomyces*, Y3 = 3 µL yeast *Saccharomyces,* Y4 = 4 µL yeast *Saccharomyces*, Y5 = 5 µL yeast *Saccharomyces*.

**Table A12:** Modeled impedance parameters Cp1, Rp1, Cp2, Rp2, Cp3, Rp3, Rs, and Ls of biochip BS9, with DI water and *S. cerevisiae.*

Circuit Element	Cp1 (F)	Rp1 (Ω)	Cp2 (F)	Rp2 (Ω)	Cp3 (Ω)	Rp3 (Ω)	Rs (Ω)	Ls (H)
BS9	5.5 × 10^−9^	1.7 × 10^4^	1.0 × 10^−9^	2.8 × 10^2^	-	-	3.6 × 10^−8^	2.4 × 10^−15^
BS9 + W20	4.1 × 10^−9^	2.4 × 10^4^	1.0 × 10^−9^	2.8 × 10^2^	3.7 × 10^−5^	4.6 × 10^1^	3.6 × 10^−8^	2.4 × 10^−15^
BS9 + W20 + Y1	2.4 × 10^−8^	1.9 × 10^3^	1.0 × 10^−9^	2.8 × 10^2^	8.8 × 10^−5^	7.0 × 10^1^	3.6 × 10^−8^	2.4 × 10^−15^
BS9 + W20 + Y2	2.6 × 10^−8^	1.8 × 10^3^	1.0 × 10^−9^	2.8 × 10^2^	9.1 × 10^−5^	6.8 × 10^1^	3.6 × 10^−8^	2.4 × 10^−15^
BS9 + W20 + Y3	2.7 × 10^−8^	1.7 × 10^3^	1.0 × 10^−9^	2.8 × 10^2^	9.3 × 10^−5^	6.7 × 10^1^	3.6 × 10^−8^	2.4 × 10^−15^
BS9 + W20 + Y4	2.8 × 10^−8^	1.5 × 10^3^	1.0 × 10^−9^	2.8 × 10^2^	9.4 × 10^−5^	6.5 × 10^1^	3.6 × 10^−8^	2.4 × 10^−15^
BS9 + W20 + Y5	2.9 × 10^−8^	1.3 × 10^3^	1.0 × 10^−9^	2.8 × 10^2^	9.5 × 10^−5^	6.3 × 10^1^	3.6 × 10^−8^	2.4 × 10^−15^

W20 = 20 µL DI water, Y1 = 1 µL yeast *Saccharomyces*, Y2 = 2 µL yeast *Saccharomyces*, Y3 = 3 µL yeast *Saccharomyces*, Y4 = 4 µL yeast *Saccharomyces*, Y5 = 5 µL yeast *Saccharomyces*.

**Table A13:** Modeled impedance parameters Cp1, Rp1, Cp2, Rp2, Cp3, Rp3, Rs, and Ls of biochip BS9, with 10% glucose as medium and *S. cerevisiae*.

Circuit Element	Cp1 (F)	Rp1 (Ω)	Cp2 (F)	Rp2 (Ω)	Cp3 (Ω)	Rp3 (Ω)	Rs (Ω)	Ls (H)
BS9	5.5 × 10^−9^	1.7 × 10^4^	1.0 × 10^−9^	2.8 × 10^2^	-	-	3.0 × 10^−8^	2.4 × 10^−15^
BS9 + G20	4.2 × 10^−9^	2.5 × 10^4^	1.0 × 10^−9^	2.8 × 10^2^	4.8 × 10^−5^	5.2 × 10^1^	3.0 × 10^−8^	2.4 × 10^−15^
BS9 + G20 + Y1	2.6 × 10^−9^	2.1 × 10^3^	1.0 × 10^−9^	2.8 × 10^2^	9.1 × 10^−5^	8.0 × 10^1^	3.0 × 10^−8^	2.4 × 10^−15^
BS9 + G20 + Y2	2.8 × 10^−9^	2.0 × 10^3^	1.0 × 10^−9^	2.8 × 10^2^	9.3 × 10^−5^	7.8 × 10^1^	3.0 × 10^−8^	2.4 × 10^−15^
BS9 + G20 + Y3	2.9 × 10^−9^	1.8 × 10^3^	1.0 × 10^−9^	2.8 × 10^2^	9.4 × 10^−5^	7.7 × 10^1^	3.0 × 10^−8^	2.4 × 10^−15^
BS9 + G20 + Y4	3.0 × 10^−9^	1.7 × 10^3^	1.0 × 10^−9^	2.8 × 10^2^	9.6 × 10^−5^	7.5 × 10^1^	3.0 × 10^−8^	2.4 × 10^−15^
BS9 + G20 + Y5	3.1 × 10^−9^	1.5 × 10^3^	1.0 × 10^−9^	2.8 × 10^2^	9.7 × 10^−5^	7.4 × 10^1^	3.0 × 10^−8^	2.4 × 10^−15^

G20 = 20 µL 10% Glucose, Y1 = 1 µL yeast *Saccharomyces*, Y2 = 2 µL yeast *Saccharomyces*, Y3 = 3 µL yeast *Saccharomyces,* Y4 = 4 µL yeast *Saccharomyces*, Y5 = 5 µL yeast *Saccharomyces*.

**Table A14:** Modeled impedance parameters Cp1, Rp1, Cp2, Rp2, Cp3, Rp3, Rs, and Ls of biochip PS9, with DI water and *S. cerevisiae*.

Circuit Element	Cp1 (F)	Rp1 (Ω)	Cp2 (F)	Rp2 (Ω)	Cp3 (Ω)	Rp3 (Ω)	Rs (Ω)	Ls (H)
PS9	6.2 × 10^−9^	1.8 × 10^3^	5.6 × 10^−9^	4.7 × 10^2^	-	-	4.4 × 10^−8^	2.4 × 10^−15^
PS9 + varing analyte	4.1 × 10^−8^	7.9 × 10^3^	5.6 × 10^−9^	4.7 × 10^2^	1.8 × 10^−5^	2.5 × 10^1^	4.4 × 10^−8^	2.4 × 10^−15^

**Table A15:** Modeled impedance parameters Cp1, Rp1, Cp2, Rp2, Cp3, Rp3, Rs, and Ls of biochip PS9, with 10% glucose as medium and *S. cerevisiae.*

Circuit Element	Cp1 (F)	Rp1 (Ω)	Cp2 (F)	Rp2 (Ω)	Cp3 (Ω)	Rp3 (Ω)	Rs (Ω)	Ls (H)
PS9	6.2 × 10^−9^	1.8 × 10^3^	5.6 × 10^−9^	4.7 × 10^2^	-	-	4.4 × 10^−8^	2.4 × 10^−15^
PS9 + varing analyte	3.6 × 10^−8^	7.3 × 10^3^	5.6 × 10^−9^	4.7 × 10^2^	2.9x × 10^−5^	3.2 × 10^1^	4.4 × 10^−8^	2.4 × 10^−15^

## Appendix B

**Table A16:** The impedance amplitude for boron doped biochip BS5 with the analyte at 40 Hz, 400 Hz, and 4 kHz (Figure A1).

	|Z|(Ω) @ 40 Hz	|Z|(Ω) @ 400 Hz	|Z|(Ω) @ 4000 Hz
BS5 + W20 + Y1	17945	14039	8009
BS5 + W20 + Y2	16287	13138	7853
BS5 + W20 + Y3	15927	12093	7466
BS5 + W20 + Y4	15690	11563	7248
BS5 + W20 + Y5	15463	10920	6952

W20 = 20 µL DI water, Y1 = 1 µL yeast *Saccharomyces*, Y2 = 2 µL yeast *Saccharomyces*, Y3 = 3 µL yeast *Saccharomyces*, Y4 = 4 µL yeast *Saccharomyces*, Y5 = 5 µL yeast *Saccharomyces.*

**Table A17:** The impedance amplitude for the biochip PS5 with the analyte at 40 Hz, 400 Hz and 4 kHz. (Figure A1).

	|Z|(Ω) @ 40 Hz	|Z|(Ω) @ 400 Hz	|Z|(Ω) @ 4000 Hz
PS5 + W20 + Y1	891	881	831
PS5 + W20 + Y2	885	877	826
PS5 + W20 + Y3	881	870	815
PS5 + W20 + Y4	876	864	806
PS5 + W20 + Y5	871	859	798

W20 = 20 µL DI water, Y1 = 1 µL yeast *Saccharomyces*, Y2 = 2 µL yeast *Saccharomyces*, Y3 = 3 µL yeast *Saccharomyces*, Y4 = 4 µL yeast *Saccharomyces*, Y5 = 5 µL yeast *Saccharomyces.*
